# Application of Aqueous Saline Process to Extract Silkworm Pupae Oil (*Bombyx mori*): Process Optimization and Composition Analysis

**DOI:** 10.3390/foods11030291

**Published:** 2022-01-21

**Authors:** Janjira Tangsanthatkun, Methavee Peanparkdee, Wattinee Katekhong, Thepkunya Harnsilawat, Chin Ping Tan, Utai Klinkesorn

**Affiliations:** 1 Department of Food Science and Technology, Faculty of Agro-Industry, Kasetsart University, 50 Ngam Wong Wan Road, Chatuchak, Bangkok 10900, Thailand; janjira_2u@hotmail.com (J.T.); methavee.pe@ku.ac.th (M.P.); fagiwnk@ku.ac.th (W.K.); 2 Research Unit on Innovative Technologies for Production and Delivery of Functional Biomolecules, Kasetsart University Research and Development Institute (KURDI), 50 Ngam Wong Wan Road, Chatuchak, Bangkok 10900, Thailand; thepkunya.h@ku.ac.th; 3 Department of Product Development, Faculty of Agro-Industry, Kasetsart University, 50 Ngam Wong Wan Road, Chatuchak, Bangkok 10900, Thailand; 4 Department of Food Technology, Faculty of Food Science and Technology, Universiti Putra Malaysia, Serdang 43400, Selangor, Malaysia; tancp@upm.edu.my

**Keywords:** silkworm pupae, oil extraction, aqueous saline method, sodium chloride concentration, response surface methodology, fatty acid profile, chemical composition

## Abstract

Silkworm pupae, a waste product from the silk production industry, can be an alternative source of edible oil, thus reducing the industry’s waste. In the present work, frozen silkworm pupae were used as raw material to extract oil via an aqueous saline process. The Box–Behnken design (BBD) and response surface methodology (RSM) were used to optimize the extraction process. The extraction conditions with the highest oil yield and a low peroxide value were obtained when using a saline solution concentration of 1.7% *w*/*v*, a ratio of aqueous liquid to silkworm pupae of 3.3 mL/g, and a 119 min stirring time at the stirring speed of 100 rpm. Under these conditions, silkworm oil with a yield of 3.32%, peroxide values of approximately 1.55 mM, and an acid value of 0.67 mg KOH/g oil was obtained. The extracted oil contained omega-3 acids (α-linolenic acid), which constituted around 25% of the total fatty acids, with approximate cholesterol levels of 109 mg/100 g oil. The amounts of β-carotene and α-tocopherol were approximately 785 and 9434 μg/100 g oil, respectively. Overall, the results demonstrated that oil extracted from silkworm pupae has good quality parameters and thus can be used as a new valuable source of edible lipids.

## 1. Introduction

Silkworm pupae are the by-product of the commercial silk industry. China, India, Uzbekistan, Thailand, and Brazil are the top five silk-producing countries in the world [1]. The desilked silkworm pupae account for 60% of the dry cocoon weight, and they are mostly used as fertilizer and animal feed, or are regarded as industrial waste [2]. Approximately 400,000 metric tons of dry silkworm cocoon are obtained from the production of 125,000 metric tons of raw silk [3]. During 2015–2020, the average annual global silk production was approximately 155,400 metric tons based on statistics from the International Sericultural Commission (https://inserco.org/en/statistics: accessed on 10 January 2022). Thus, approximately 497,300 metric tons of dry cocoon were produced, which equates to approximately 298,400 metric tons of desilked pupae each year. Recently, the use of agricultural waste and the management of industry by-products have become a focus of research as we seek to curb climate change and minimize our impact on the Earth [4]. With this in mind, the proper utilization of silkworm pupae could not only generate extra income for farmers in the silk industry but could significantly reduce the industry’s inherent waste. In Thailand, silkworms are classified as mulberry (*Bombyx mori*) and non-mulberry or eri silkworm (*Samia ricini*), each of which has different nutritional value. Two main nutrients in the pupae of silkworms are protein and oil, which vary in the range of 48–67 wt% and 17–30 wt% (dry basis), respectively [5].

Oil extracted from silkworm pupae has been reported as a safe oil source and nutritionally equivalent to commonly used vegetable oils, such as sunflower oil [6]. This oil is a source of unsaturated fatty acids (approximately 60–70% of the total fatty acid content), particularly α-linolenic and oleic acids. Both α-linolenic and oleic acids are known for their nutritional and health benefits, which could be exploited for various applications, including food, supplements, and feed [2,7,8,9]. To separate oil from the silkworm pupae, several techniques were previously used, including mechanical pressing extraction [10] and solvent and supercritical fluid extraction [2,6,11,12,13,14]. Using these methods requires the silkworm pupae to be dry before the extraction can be initiated, and the extraction conditions may alter the quality of the protein co-products. Due to its safe nature and limited environmental impact, aqueous oil extraction can be used as an alternative method for the extraction of oil from silkworm pupae.

In the aqueous extraction method of lipids from insects or the muscle tissue of other organisms (e.g., fish), mincing, grinding, or homogenizing is usually done in the procedure’s early steps. Next, the lipids in grounded or homogenized tissues are then extracted with an aqueous solution using tissue hydrolysis with enzymes, alkaline, or acids to improve the extraction efficiency [15,16,17,18,19]. However, using a hydrolysis reaction limits the potential reuse of protein components and regularly results in a bitter taste [20,21]. Moreover, issues have been reported with attempts to scale up the enzymatic hydrolysis method [22]. In previous studies, common salt or sodium chloride has been shown to enhance the oil extraction from olive paste and fish tissue by increasing the gradient of ionic charge and density between the oil and hydrophilic phases [22,23]. Using an aqueous saline extraction process to extract the oil from silkworm pupae has not yet been investigated and could prove to be a valuable extraction method.

This study aimed to develop an aqueous saline method for extracting oil from silkworm pupae. The main variables investigated in the use of this method included determining the ideal sodium chloride concentration, aqueous liquid-to-solid (silkworm) ratio, and stirring time. Response surface methodology (RSM) is an optimal tool for process optimization. Hence, we used RSM to optimize the extraction conditions in order to enhance the production yield and the quality of the silkworm oil. In addition, the fatty acid composition and other components, including cholesterol, α-tocopherol, and β-carotene, of the extracted silkworm oil were also analyzed.

## 2. Materials and Methods

### 2.1. Raw Materials and Chemicals

Frozen mulberry desilked silkworm pupae (*Bombyx mori*), used in all the experiments, were obtained from a local silkworm farm in Phetchabun Province, Thailand. The silkworm pupae samples were kept at approximately −18 °C until the experiment was performed. Deionized water (electricity resistivity ≥ 15 MΩ.cm) was used for the preparation of all the solutions. Sodium chloride (NaCl), phenolphthalein (C_20_H_14_O_4_), barium chloride (BaCl_2_), iron (II) sulfate (FeSO_4_), and ammonium thiocyanate (NH_4_SCN) were purchased from Ajax Finechem Pty, Ltd. (New South Wales, Australia). Hexane (C_6_H_14_) and butanol (C_4_H_10_O) were purchased from RCI Labscan, Ltd. (Bangkok, Thailand), while isopropyl alcohol (2-propanol) was purchased from J.T. Baker (Avantor Performance Materials, LLC, Allentown, PA, USA). Toluene (C_7_H_8_), potassium hydroxide (KOH), and methanol (CH_3_OH) were supplied by Merck KGaA (Darmstadt, Germany). Cumene hydroperoxide (C_6_H_5_C(CH_3_)_2_OOH) and 2,2-diphenyl-1-picrylhydrazyl (DPPH) were purchased from Sigma-Aldrich Co. (St. Louis, MO, USA). All the chemicals used were reagent-grade.

### 2.2. Extraction of the Silkworm Pupae Oil

The aqueous saline extraction process was carried out according to the method of Tzompa-Sosa et al. [19] and Kadioglu et al. [24], with some modifications. To extract the oil, 100 g of each frozen silkworm pupae was mixed with a saline (NaCl) solution of a specified concentration and volume. Then, the mixture was blended for 3 min using a food processor (Phillips HR2118, Phillips International Co., Ltd., Samut Prakan, Thailand), followed by stirring at 100 rpm with an overhead stirrer (IKA^®^ Rw 20 digital, Staufen, Germany) for a specified time. Next, the suspension was passed through a 45-mesh stainless-steel screen to eliminate large particles (>354 μm). The obtained silkworm pupae suspension was then centrifuged (Sorvall RC6 plus, Thermo Fisher Scientific, Karlsruhe, Germany) at 10,000 rpm (~15,180× *g*) for 1 h at 25 °C to obtain three fractions, including a cream layer, a supernatant or an aqueous layer, and a pellet from top to bottom, respectively. The top cream layer and supernatant phase were collected and subsequently centrifuged using a high-speed, refrigerated microcentrifuge (MX-307, TOMY Digital Biology Co., Ltd., Tokyo, Japan) at 15,000 rpm (~20,380× *g*) for 20 min at 25 °C. This second centrifugation was performed at a higher speed than the first one to enhance lipid separation. After the second centrifugation, free oil was obtained in the upper layer of the centrifuge tube. The lower layers contained a thin emulsion layer, a clear supernatant, and a residue. The transparent oil and emulsion layers were then pipetted and mixed with hexane (30 mL). The extracted oil was separated by an evaporating solvent using a parallel evaporator equipped with a vacuum controller (Buchi Syncore^®^, BÜCHI Labortechnik AG Co., Flawil, Switzerland) at 50 °C and 250 mbar. Nitrogen flushing was also used to ensure that there was no solvent remaining in the extracted oil and to replace the oxygen, which can affect the quality of the oil. The amount of extracted oil was weighed to calculate the extraction yield by following Equation (1). The extracted oil for each treatment was kept in an amber glass bottle and stored at approximately −18 °C for further analysis.

(1) Extraction yield (%)=(Oil weight/Sample weight)×100

### 2.3. Determination of the Peroxide Value (PV)

The PV of the silkworm pupae oil was analyzed by following the lipid peroxide assay adapted from Prichapan et al. [25]. Briefly, 1 mL of a barium chloride solution (0.132 M BaCl_2_ in 0.4 N HCl) was added to 1 mL of an iron sulfate solution (0.144 M FeSO_4_), then vortexed for 1 min, and centrifuged (Gallenkamp Junior Centrifuge, Gallenkamp & Co., Ltd., London, UK) at a maximum speed of 4460 rpm (~2450× *g*) for 3 min. Next, 1 mL of the clear supernatant was taken and mixed with 1 mL of an ammonium thiocyanate solution (3.94 M NH_4_SCN) to obtain the ferrous reagent. Next, 30 μL of this reagent solution was added to the oil sample solution, which was previously prepared by dissolving 0.2 mL oil in a 2.8 mL methanol/butanol mixed solution (2:1 *v*/*v*). The sample solution was kept in the dark at room temperature for 20 min, and the absorbance of the resulting solution was then measured at a wavelength of 510 nm using a UV–Vis spectrophotometer (Genesys 20, Thermo Spectronic, Waltham, MA, USA). The lipid hydroperoxides or PV in mM were calculated using the standard curve of cumene hydroperoxide.

### 2.4. Determination of the Acid Value (AV)

The AV of the silkworm pupae oil was determined by following the AOCS Cd-3d-63 method [26], where 1 g of oil was dissolved in an isopropyl alcohol and toluene solution (1:1 *v*/*v*). After mixing, the sample solution was titrated using a 0.01 N potassium hydroxide (KOH) solution and phenolphthalein as an indicator. The exact volume of the titrant was recorded when the end point (pink solution) was reached. The AV in mg KOH/g oil was expressed after calculations according to the following equation:

(2) AV = [(A−B)×N ×56.1]/W

where A and B represent the mL of KOH solution used in the titrating sample and the blank, respectively. N is the normality of the KOH solution, and W is the mass of the oil sample (g).

### 2.5. Analysis of the Chemical Composition of the Silkworm Pupae and Oil

The chemical composition of the silkworm pupae, including the moisture, total protein, total fat, total carbohydrate, dietary fiber, and ash, was determined according to the method of the Association of Official Analytical Chemists (AOAC). The fatty acid composition of the oil was analyzed using gas–liquid chromatography (GLC) according to the in-house method based on the official AOAC 963.22 and AOAC 969.33 methods. The standard AOAC 994.10 analytical method was used to analyze the cholesterol content [27]. To determine the α-tocopherol and β-carotene content, in-house methods using high-performance liquid chromatography (HPLC) were applied [28,29].

### 2.6. Determination of 2,2-Diphenyl-1-Picrylhydrazyl (DPPH) Free Radical Scavenging Activity

The DPPH radical scavenging activity of silkworm oil was determined using the method of Singh et al. [30], with slight modifications. An aliquot (100 μL) of oil was added to 4.9 mL of a 0.1 mM DPPH solution in methanol. The solution was then vortexed vigorously and left for 30 min in the dark under ambient conditions. The absorbance was measured at 517 nm using a UV–Vis spectrophotometer (Genesys 20, Thermo Spectronic, Waltham, MA, USA). The absorbance of each sample was recorded, and the percentage inhibition of DPPH was calculated according to the following formula:

(3) $$\mathrm{Inhibition}\;\mathrm{percentage}\;\left(\%\right)=100\times \left(A_{B}-A_{S}\right)/A_{B}$$

where A_B_ and A_S_ are the absorbance values of the blank (methanol) and of the tested samples, respectively.

### 2.7. Experimental Design and Statistical Analysis

Three independent variables, namely the concentration of NaCl (X_1_, % *w*/*v*), the liquid-to-solid ratio (X_2_, mL/g), and the stirring time (X_3_, min), were investigated for the aqueous saline extraction of silkworm pupae oil. In the Box–Behnken design (BBD), three levels for each variable were applied according to the single-factor experimental results. The optimal combination of independent variables in oil extraction based on the extracted oil yield (Y_1_) and PV (Y_2_) as response variables was acquired using RSM. To check the variability of the RSM experiment, the central points were repeated three times. All experiments were carried out twice and the full second-order polynomial regression model was developed as follows:

(4) $$Y={\;\beta }_{0}+\overset{3}{\underset{i=1}{\sum }}\beta_{i}X_{i}+\overset{3}{\underset{i=1}{\sum }}\beta_{\mathrm{ii}}X_{i}^{2}+\overset{3}{\underset{i\neq j=1}{\sum }}\beta_{\mathrm{ij}}X_{i}X_{j}$$

where Y represents the response variable, β_0_ is the regression intercept, and β_i_, β_ii_, and β_ij_ are the regression coefficients for the linear, quadratic, and interactive effects of the model, respectively. X_i_ and X_j_ are the independent variables [31].

An analysis of variance (ANOVA) was performed to determine the lack of fit and the effect of the linear, quadratic, and interaction terms of each of the response variables. The analysis of the experimental data and the optimization process were carried out using Minitab Statistical Software Cloud-Based Version (Minitab LLC., State College, PA, USA). Differences between the means were analyzed based on a significance level of *p* < 0.05, using IBM SPSS Statistics Version 28.0 (Thaisoftup Co., Ltd., Bangkok, Thailand).

## 3. Results and Discussion

### 3.1. Chemical Composition of Silkworm Pupae

The nutrient compositions of the silkworm pupae are presented in Table 1. In fresh samples, silkworm pupae had a protein content of approximately 11.7%, which is higher than the 10.3% protein content reported for the edible insect *Acheta domesticus* [32], but not as high as the 16% and 24% protein content reported for the eri silkworm pupae grown on tapioca and castor leaves, respectively [33], and the 24.4% protein content of another edible insect, *Euxoa auxiliaries* [34]. Based on the definition of FAO/WHO, the majority of insects and pupae are regarded as high in protein (over 10 g proteins/100 g edible portion of fresh weight basis) [35]. A fat content of around 6.1% was observed in the silkworm pupae used in this study, which was lower than that found in eri silkworm pupae (8.0–8.6%) and reported by Longvah et al. [33]. Furthermore, the oil content of the silkworm *Bombyx mori* in the pupal stage was different between fresh male (4.8%) and female (9.0%) pupae [10]. Total carbohydrate and dietary fiber (~4.0% and 1.1%, respectively) comparable to the values reported by Longvah et al. were also found in the silkworm pupae [33]. The silkworm pupae had an energy of approximately 521 kcal/100 g dried pupae, which is in the range of the caloric values for insects and pupae (293–762 kcal/100 g dry weight) as reported by Rumpold and Schlüter [36]. These values depend on the insects’ diet, species, and life stage. Two major nutrients, protein and fat (~51.9% and 26.9%, respectively), were consistent with what was reported in previous works [7,33]. In general, insects and pupae are composites containing proteins, lipids, and polysaccharides. Silkworm pupae have been reported to be 30–55% protein, 10–15% carbohydrates, and 25–30% oil, which is rich in various saturated and unsaturated fatty acids [37,38,39,40,41]. The high protein and fat content of silkworm pupae indicate their extensive potential as an alternative source for food and feed.

### 3.2. Effect of a Single Factor on the Extraction Yield

#### 3.2.1. Effect of Stirring Time

**Figure 1:**
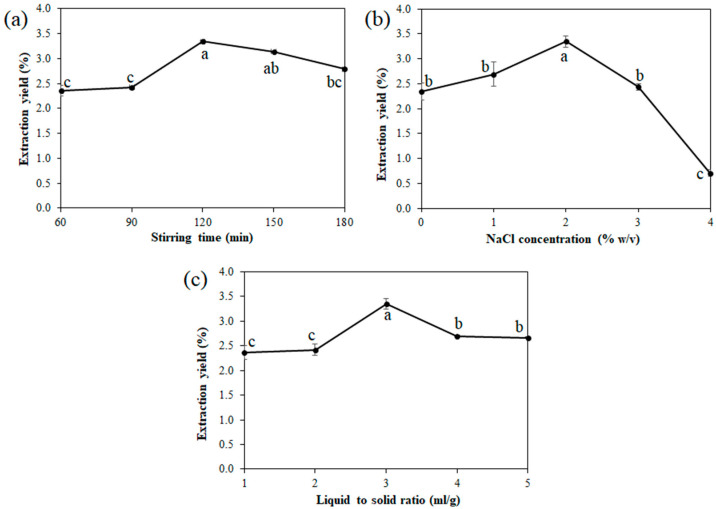
The effect of stirring time (**a**), NaCl concentration (**b**), and liquid-to-solid ratio (**c**) on the extraction yield of silkworm pupae oil. Different letters indicate significant differences (*p* ≤ 0.05).

Figure 1a shows the effect of stirring time on the extraction yield. The results indicate a significant increase in the extraction yield after increasing the stirring time from 60 min (2.35% ± 0.05%) to 120 min (3.35% ± 0.11%) and a slight decrease with an additional increase in stirring time to 180 min (2.79% ± 0.40%) (*p* ≤ 0.05). The reason for this could be that a longer stirring time leads to the sample’s emulsification, which may then trap the oil in the system and result in a decline in the oil’s yield [19,42,43]. Therefore, a longer stirring time is unnecessary once the maximum extraction yield is achieved. Based on the results, stirring time should be controlled in the range of 120 min, which is sufficient to attain the extraction yield for this treatment.

#### 3.2.2. Effect of the Concentration of NaCl

The silkworm pupae oil was extracted with various concentrations of NaCl by an aqueous extraction method. As shown in Figure 1b, the extraction yields positively correlated with increasing concentrations of NaCl from 0% to 2% *w*/*v* and reached the highest value of 3.35% ± 0.11% when using 2% *w*/*v* NaCl. This value significantly decreased when using NaCl above this concentration (*p* ≤ 0.05). This could be because inorganic salt increases the polarity difference between oil and water, thereby reducing the oil’s solubility in the water and enhancing the process’s salt-assisted effect [44]. In other words, a higher concentration of NaCl can cause a salting-out effect that leads to protein aggregation and precipitation. This phenomenon will result in the trapping of oil by protein, which will restrict the release of oil and lower the yield of extracted oil [43,45,46]. Hashemi et al. [47] reported that the addition of NaCl also decreased the extraction time and reduced the required temperature for the extraction of *Vitex pseudonegundo* by ohmic-assisted hydrodistillation. However, when increasing the saline solution above 2% *w*/*v*, the extraction yields significantly decreased (*p* ≤ 0.05), and it should be noted that protein denaturation affected the oil extraction [48].

#### 3.2.3. Effect of the Liquid-to-Solid Ratio

The liquid-to-solid ratio is an important factor that can influence the extraction efficiency. Different liquid-to-solid ratios (1–5 mL/g) were determined, and the results are presented in Figure 1c. It was found that the extraction yield tended to increase with increasing liquid saline volume, beginning with 2.37% ± 0.13% at a liquid-to-solid ratio of 1 mL/g and reaching a maximum extraction yield of 3.35% ± 0.11% at a liquid-to-solid ratio of 3 mL/g. After this point, the extraction yields moderately decreased and remained constant (~2.70%) with a continuous increase in the ratio from 4 to 5 mL/g (*p* ≤ 0.05). This may be because the oil–water separation effect was minimized with a high ratio of water to material [44]. The results suggested that a suitable amount of saline solution can easily achieve the maximum extraction yield of the target compounds. Although a large amount of extraction solvent can increase the leaching-out rates of target compounds, it also leads to the waste of extraction solvent and an extra layer of complexity in the extraction procedure [49].

### 3.3. Effect of Independent Processing Parameters on Response Variables from the BBD Experiment

The effect of different levels of the independent variables, including the NaCl concentration, liquid-to-solid ratio, and stirring time, on the yield (Y_1_), PV (Y_2_), and AV (Y_3_) of silkworm pupae oil was determined at different treatment combinations, and all the results are summarized in Table 2. The yield of extracted silkworm pupae oil varied in the range of 2.39–3.50 wt%, with a mean value of ~2.82 wt%, or accounting for ~54.7% recovery oil (Equation (S1)). The differences in the fat content of the silkworm pupae oil varied according to its species, origin, and metamorphosis stage and the extraction’s method and conditions [36,50,51,52]. For example, Shanker et al. [52] studied the characteristics of the neutral lipid of desilked eri silkworm pupae (*Samia cynthia ricini*) fed with castor (*Ricinus communis Linn*.) and tapioca (*Manihot utilissima Pohl*.) leaves. The oil content in the pupae was in the range of 18–20% (dry basis). In addition, Longvah et al. [33] reported that the fat content observed in eri silkworm prepupae grown on either castor or tapioca was not different (~26.2%), while the eri silkworm pupae grown on caster had higher fat content than those grown on tapioca (25%) (dry basis). The fat content tended to be higher during the stages of pupae, larvae, and wintering than during other periods for the same species [51].

The PV and AV are important quality parameters related to the extent of the oil’s oxidation and hydrolysis, respectively. The statistical analysis of the results showed that the PV of extracted oil was significantly dependent on the extraction conditions (*p* ≤ 0.05). However, the observed values varied from 1.40 to 2.10 mM (Table 2) and fell within the suggested value of ~6.69 mM or 15 meq/kg oil (Table S1) for virgin oils [53]. The AV is responsible for the release of free fatty acids. In this study, the AV of oil varied from 0.53 to 0.75 mg KOH/g oil (Table 2). These values are lower than the maximum recommended value of 4 mg KOH/g for virgin fats and oils [53]. However, there were no significant (*p* > 0.05) effects of the extraction conditions on the AV of extracted oils. This means that the NaCl concentration, liquid-to-solid ratio, and stirring time did not affect the acidity of the silkworm pupae oil.

### 3.4. Model Fitting

A multiple regression analysis was performed based on the experimental data (Table 2), and the backward second-order polynomial regression models as a function of independent variables were established for the extracted yield (Y_1_) and PV (Y_2_) of the silkworm pupae oil. The estimated coefficients of the independent variables are shown in Table 3, and the equations in terms of actual levels are presented as follows:

(5) $$Y_{1}=-5.650+2.186X_{1}+1.691X_{2}+0.070X_{3}-0.567X_{1}^{2}-0.249X_{2}^{2}-0.0003X_{3}^{2}$$

(6) $$Y_{2}=4.048+0.088X_{1}-1.620X_{2}+0.247X_{2}^{2}$$

The adequacy and predictability of the models were considered according to the determination coefficient (R^2^), adjusted determination coefficient (R^2^_adj_), lack of fit, and absolute average deviation (AAD) [31]. For the extracted oil yield, the R^2^ and R^2^_adj_ were 0.948 and 0.908, respectively (Table 3), suggesting the model’s excellent fitting degree, i.e., it could interpret more than 90% of the variation in the response variables studied. Considering the PV results in which the R^2^ and R^2^_adj_ were 0.704 and 0.623, respectively, we observed a good fitting degree of the model, which could interpret more than 62% of the variation in the response variables studied [54]. To support the adequacy and how well the model fitted the data, a lack-of-fit test was used. In this work, *p*-values of the model’s “lack of fit” were 0.480 and 0.802 for the extracted yield and PV of the silkworm pupae oil, respectively. These values suggested that the “lack of fit” had no statistical significance (*p* > 0.05), which verified the reliability of the models [55].

**Figure 2:**
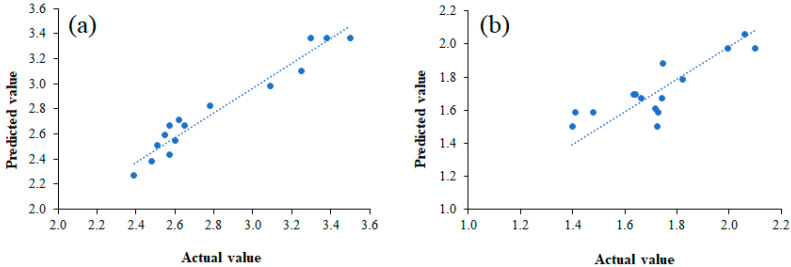
The correlation plots of actual and predicted values of the extraction yield (**a**) and peroxide value (**b**).

**Figure 3:**
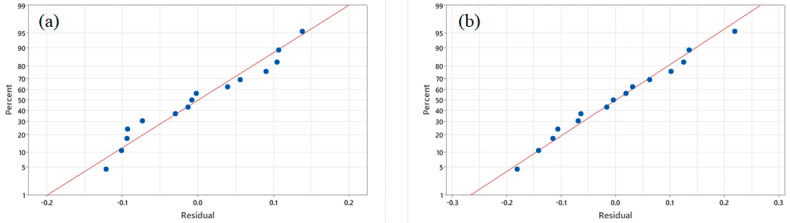
The plots of normal probability of residuals for the extraction yield (**a**) and peroxide value (**b**).

Furthermore, AAD, another realized parameter, was also calculated to measure the models’ adequacy and accuracy [31]. The suggested range of 0% to 30% for AAD was reported in a previous work by Bas and Boyaci [56]. For the present study, the AAD values obtained for the oil yield and PV were small, with values of 2.76% and 5.58%, respectively (Table 3). These small values confirmed the satisfactory fit of the regression models to the experimental data. This was consistent with the correlation plots of the experimental or actual and predicted values (Figure 2). A linear distribution was observed for both the oil yield and PV data, indicating well-fitting models (R^2^ ~0.999 and 0.996, respectively). The normal probability plot is also presented in Figure 3 to show the normality of the internally studentized residual. The plot indicated that the residuals (the difference between the actual and predicted values) followed a normal distribution and formed an approximately straight line, and the fitted model provided a reasonable estimate for oil extraction yields. According to the criteria discussed above, these results indicated that the regression models are adaptable and can be used as response surface models for an estimation of the mean response.

### 3.5. Analysis of the Response Surface

**Figure 4:**
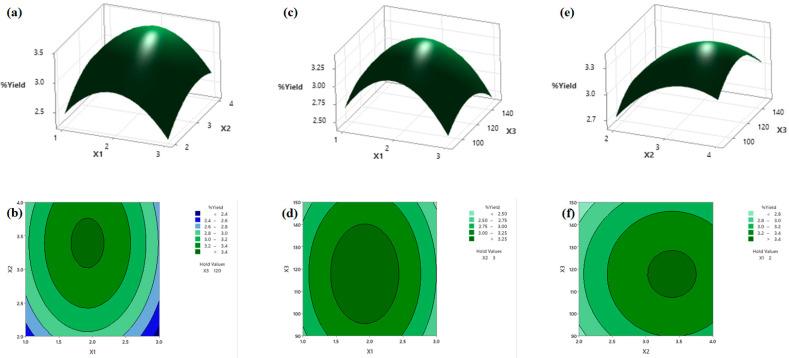
Response surface (**a**,**c**,**e**) and contour plots (**b**,**d**,**f**) as a function of independent variables on the extraction yield. (**a**,**b**) NaCl concentration and liquid-to-solid ratio, (**c**,**d**) NaCl concentration and stirring time, (**e**,**f**) stirring time and liquid-to-solid ratio. X_1_, X_2_, and X_3_ represent the NaCl concentration, liquid-to-solid ratio, and stirring time, respectively.

**Figure 5:**
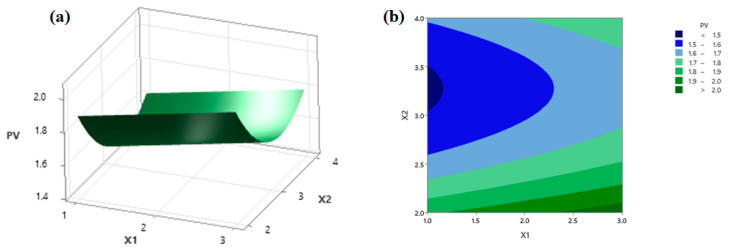
The response surface (**a**) and contour plots (**b**) as a function of independent variables on the PV. X_1_ and X_2_ represent the NaCl concentration and liquid-to-solid ratio, respectively.

The interaction between the response and the independent variables was visually interpreted using a response surface and contour plots. The plots represented the response level against two independent variables at a central level of the remaining independent variable. The response surface and contour plots of the extracted yield of silkworm pupae oil are shown in Figure 4 for the interaction between the NaCl concentration and liquid-to-solid ratio (Figure 4a,b), NaCl concentration and stirring time (Figure 4c,d), and liquid-to-solid ratio and stirring time (Figure 4e,f). Similarly, these plots between NaCl concentration and liquid-to-solid ratio for the PV of extracted oil are also shown in Figure 5. Figure 4a,b indicate the interaction effect between the NaCl concentration and the liquid-to-solid ratio on the oil yield extracted at a 120 min stirring time. The oil yields gradually increased with an increase in NaCl concentration from 1 to 2% *w*/*v* at the designated liquid-to-solid ratio and then decreased with an increase in the concentration of NaCl. An increase in the concentration of NaCl has advantages for extraction because an increase in the polarity of this solution leads to a decrease in the solubility of nonpolar compounds, thus resulting in a high extracted yield [46]. The disadvantage of using excess NaCl concentrations is a salting-out effect, which promotes the aggregation and precipitation of proteins and affects the extraction process [57]. Similarly, the oil yields obviously increased and then decreased with the increase in the liquid-to-solid ratio at any NaCl concentration. These results are probably due to the decrease in the oil–water separation effect because of the high ratio of water to material [45].

Figure 4c,d represent the interaction effect between the NaCl concentration and stirring time on the oil yield extracted at a liquid-to-solid ratio of 3 mL/g. An increase in the stirring time improved the oil yields to some degree at any NaCl concentration, but a further increase in the stirring time caused a decrease in oil yields. A similar effect in the stirring time as shown in Figure 4e,f was also observed, indicating an interaction effect between the liquid-to-solid ratio and the stirring time on the oil yield extracted at 2% *w*/*v* of NaCl concentration. An increase in the oil yield was seen when the stirring time was increased from 90 to 120 min. However, the oil yield tended to decrease with longer stirring times from 120 to 150 min. This effect was observed for all levels of liquid-to-solid ratios used in this study. The increase in oil yields with increasing stirring times was mainly ascribed to the fact that a longer stirring time could facilitate the oil transfer from the ruptured pupae tissue particles to the aqueous phase. This trend was also observed in a recent study, where the extraction time had a positive effect on the oil yield [45,58].

Regarding the PV, the response surface and contour plots (Figure 5a,b, respectively) as a function of the NaCl concentration and the liquid-to-solid ratio at a 120 min stirring time were established according to the significant terms in the regression model; see Equation (6). It showed that the PV of silkworm pupae oil was positively influenced by the NaCl concentration at any liquid-to-solid ratio. The results indicated that the PV of silkworm pupae oil was greater at both low and high levels of liquid-to-solid ratios at the designated NaCl concentration. At an intermediate level of the liquid-to-solid ratio, however, the PV of extracted oil displayed the lowest value (less than 1.5 mM). The increase in PV with an increase in NaCl concentration may be because NaCl promotes peroxide formation, leading to an increase in peroxide values. The presence of NaCl is known to increase oxidation since it can readily donate its valence electron [59,60].

### 3.6. Optimization and Validation of the Regression Model

Response optimization was performed to analyze the optimal levels of independent variables required to achieve the maximum extraction yield and minimum PV of silkworm pupae oil. To determine the exact levels for all the independent variables necessary to achieve the optimal conditions, a numerical optimization was utilized. Results showed that the extraction using a 1.707% *w*/*v* NaCl solution at a 3.273 mL/g liquid-to-solid ratio and a 119.307 min stirring time provided the optimal maximum extraction yield and minimum PV with a desirability of 1000 (Table 4). After considering the feasibility of these parameters during real production, the optimum conditions were modified to an NaCl solution of 1.7% *w*/*v*, a liquid-to-solid ratio of 3.3 mL/g, and a stirring time of 119 min. Under these conditions, the predicted maximum yield and minimum PV of silkworm pupae oil were 3.38% and 1.54 mM, respectively. Using these same conditions, the average values of the extraction yield and PV of silkworm pupae oil obtained from the experiment were 3.32% ± 0.03% and 1.55 ± 0.02 mM, respectively. These experimental results corresponded fairly well to the predicted values, which were not significantly different (*p* > 0.05) from the predicted values (Table 4). The relative error values between the predicted and actual oil yield and PV were also calculated as 1.81% and 0.65%, respectively. This revealed that the models were well fitted for the extraction of oil from silkworm pupae under optimal aqueous saline conditions, and the designed models were good for predicting the optimal extraction conditions.

### 3.7. Chemical Compositions of Silkworm Pupae Oil Extracted by Aqueous Saline

#### 3.7.1. Fatty Acid Composition

The fatty acid composition of the silkworm pupae oil obtained from aqueous saline extraction is presented in Table 5. The most abundant fatty acid in the extracted silkworm pupae oil was oleic acid (C18:1, 36.84 ± 0.03), which generally exists as a monounsaturated fatty acid (MUFA) in most edible fats and oils. Silkworm pupae oil is a source of essential polyunsaturated fatty acids (PUFAs) named α-linolenic (C18:3, 24.85 ± 0.06) and linoleic acid (C18:2, 4.25 ± 0.01), which belong to n-3 (omega-3) and n-6 (omega-6) series fatty acids, respectively. The total saturated fatty acids found in the extracted silkworm pupae oil accounted for approximately 33% of the total fatty acids. Palmitic (C16:0, 26.0% ± 0.05%) and stearic acids (C18:0, 6.78 ± 0.01) were the main acids found in the extracted oil. The composition of fatty acid observed in the silkworm pupae oil corresponded with previously published data [7,8]. However, it is important to note that the higher and lower ratios in the fatty acid composition are due to differences in extraction methods and factors, species’ origin, sex, maturation stage, season, and geographical regions, as reported in previous studies [2,9].

#### 3.7.2. Minor Components

As shown in Table 5, the cholesterol level in the extracted silkworm pupae oil was 108.7 mg/100 g, which is comparable to beef tallow [61,62] and slightly higher than is seen in lard and chicken fat [61]. However, the cholesterol level of the silkworm pupae oil was lower than those found in fish oils and milk fat [62,63,64]. The cholesterol content in extracted silkworm pupae oil was lower than what was reported by Belluco et al. and Winitchai et al. [65,66]; it has been shown that *B. mori* chrysalises contain a high concentration of cholesterol (214 mg/100 g) [60]. In addition, it was found that the average cholesterol content in four types of insects’ lipids including termites’ (*Macrotermes bellicosus*), caterpillars’ (*Imbrasia belina*), and beetle larvae of *Oryctes rhinoceros* and *Rhynchophorus phoenicis* were around 3.6% [67].

The contents of naturally occurring antioxidants, especially carotenoids and tocopherols, are the most important factor affecting the oxidative stability of edible oils. Therefore, the amount of β-carotene and α-tocopherol in the extracted silkworm pupae oil was determined and found to be approximately 785 and 9434 μg/100 g oil, respectively. Regarding the content of β-carotene, it was found to be lower than the content of crude palm oil [68] but higher than the content of crude rapeseed and sunflower oils [69], cold-pressed hemp oil, flax oil, and canola seed oil [70]. Regarding α-tocopherol, the silkworm pupae oil extracted by the aqueous saline method in our study contained less α-tocopherol than some crude and refined vegetable oils [71,72]. However, it was higher than the values reported for cold-pressed hemp and flax seed oils [70]. Higher and lower values of the α-tocopherol content of silkworm pupae oil had been reported in previous studies and are agreed to be due to differences in sex, maturation stage, and extraction method [11,66,73].

### 3.8. DPPH Free Radical Scavenging Activity

Lipid oxidation accelerates when free radicals form as a result of unsaturated fatty acids losing hydrogen atoms from their double bonds [74]. The scavenging of free radicals initiates the oxidation mechanism and inhibits the chain reaction, thus preventing lipid oxidation [75]. The DPPH free radical scavenging effect of silkworm pupae oil was 57.59 ± 0.93%. This result was comparable to a previous study revealing the antioxidant activity of the muga silkworm (*Antheraea assamensis*) [76] and suggested that silkworm pupae oil had a noticeable effect on scavenging DPPH free radicals. In general, silkworm pupae oil contains tocopherols, natural phenolic antioxidants that are good sources of vitamin E and play an important role as lipid oxidation inhibitors [11]. The results demonstrated that the extracted silkworm pupae oil had good quality parameters, making it a useable and valuable source of edible lipids.

## 4. Conclusions

This study identified that aqueous saline extraction is an efficient, green, and simple method for silkworm oil extraction. An RSM-based BBD was successfully used to evaluate the influence of independent variables, including the NaCl concentration, aqueous liquid to silkworm ratio, and extraction time, on the oil’s yield and quality. The optimal conditions of the extraction process were found to be 1.7% *w*/*v* NaCl, a 3.3 mL/g liquid-to-solid ratio, and a 119 min extraction time. Under these extraction conditions, the highest yield (3.32 wt%) and highest-quality oil (PV and AV of 1.55 mM and 0.67 mg KOH/g oil, respectively) were obtained. Overall, the analyzed results of the fatty acid composition and the content of minor components demonstrated that silkworm oil obtained from this alternative extraction method has the potential to be used as a source of edible oil in the food industry. The comparison of the extraction efficiency, quality, composition, and nutritional values of this oil with other extraction methods such as mechanical, solvent extraction, and supercritical fluid will be carried out in a separate study.

## Figures and Tables

**Table 1:** The nutrient composition of silkworm pupae.

Nutrient	Content (per 100 g)	
	Fresh Pupae	Dry Basis
Moisture (g)	77.43 ± 0.08	-
Energy (kcal)	117.55 ± 0.49	520.69 ± 3.96
Protein (g)	11.72 ± 0.01	51.89 ± 0.15
Total fat (g)	6.07 ± 0.15	26.87 ± 0.75
Total carbohydrate (g)	4.03 ± 0.21	17.83 ± 0.85
Dietary fiber (g)	1.07 ± 0.06	4.72 ± 0.30
Ash (g)	0.77 ± 0.01	3.41 ± 0.05

**Table 2:** The experimental run and extraction conditions for the Box–Behnken design (BBD) with coded levels of each variable (blanket), including the response values for the extraction yield, PV, and AV of silkworm pupae oil.

Run	NaCl Concentration (% *w*/*v*, X_1_)	Liquid-to-Solid Ratio (mL/g, X_2_)	Stirring Time (min, X_3_)	Extraction Yield (%, Y_1_)		PV (mM, Y_2_)		AV (mg KOH/g, Y_3_)
				Exp	Pred	Exp	Pred	
1	2 (0)	4 (1)	90 (−1)	3.25 ± 0.02 ^c^	3.10	1.64 ± 0.03 ^cd^	1.70	0.61 ± 0.09 ^a^
2	2 (0)	4 (1)	150 (1)	3.09 ± 0.05 ^d^	2.98	1.64 ± 0.05 ^cd^	1.70	0.56 ± 0.14 ^a^
3	3 (1)	3 (0)	90 (−1)	2.51 ± 0.02 ^gh^	2.51	1.66 ± 0.03 ^cd^	1.68	0.72 ± 0.10 ^a^
4	1 (−1)	4 (1)	120 (0)	2.78 ± 0.01 ^e^	2.83	1.72 ± 0.19 ^cd^	1.61	0.64 ± 0.15 ^a^
5	3 (1)	4 (1)	120 (0)	2.57 ± 0.03 ^fg^	2.67	1.82 ± 0.14 ^bc^	1.78	0.75 ± 0.18 ^a^
6	3 (1)	3 (0)	150 (1)	2.48 ± 0.04 ^gh^	2.33	1.74 ± 0.12 ^c^	1.68	0.71 ± 0.11 ^a^
7	1 (−1)	3 (0)	150 (1)	2.60 ± 0.06 ^fg^	2.55	1.40 ± 0.03 ^e^	1.50	0.64 ± 0.15 ^a^
8	1 (−1)	3 (0)	90 (−1)	2.65 ± 0.01 ^f^	2.67	1.72 ± 0.24 ^cd^	1.50	0.53 ± 0.15 ^a^
9	1 (−1)	2 (−1)	120 (0)	2.57 ± 0.03 ^fg^	2.44	1.74 ± 0.15 ^c^	1.88	0.64 ± 0.15 ^a^
10	2 (0)	3 (0)	120 (0)	3.30 ± 0.04 ^bc^	3.37	1.41 ± 0.03 ^e^	1.59	0.68 ± 0.08 ^a^
11	2 (0)	2 (−1)	90 (−1)	2.62 ± 0.11 ^fg^	2.71	1.99 ± 0.05 ^ab^	1.97	0.64 ± 0.15 ^a^
12	2 (0)	3 (0)	120 (0)	3.50 ± 0.09 ^a^	3.37	1.73 ± 0.08 ^cd^	1.59	0.72 ± 0.10 ^a^
13	2 (0)	2 (−1)	150 (1)	2.55 ± 0.08 ^gh^	2.59	2.10 ± 0.10 ^a^	1.97	0.55 ± 0.06 ^a^
14	3 (1)	2 (−1)	120 (0)	2.39 ± 0.02 ^h^	2.27	2.06 ± 0.10 ^a^	2.06	0.63 ± 0.09 ^a^
15	2 (0)	3 (0)	120 (0)	3.38 ± 0.04 ^ab^	3.37	1.48 ± 0.22 ^de^	1.59	0.62 ± 0.08 ^a^

Mean values followed by different letters in the same column indicate significant differences (*p* ≤ 0.05). Exp and Pred represent experimental and predicted values, respectively.

**Table 3:** The estimated coefficient of the independent variables with the associated statistical significance of each coefficient for backward regression models.

Regression Terms ^a^	Extraction Yield (%, Y_1_)			PV (mM, Y_2_)		
	Coefficient	F-Value	*p*-Value ^b^	Coefficient	F-Value	*p*-Value ^b^
Model		24.17	0.000		8.71	0.003
Constant (β_0_)	−5.650			4.048		
X_1_ (β_1_)	2.186	4.04	0.079	0.088	3.71	0.080
X_2_ (β_2_)	1.691	23.57	0.001	1.620	8.75	0.013
X_3_ (β_3_)	0.070	0.93	0.363			
X_1_X_1_ (β_11_)	−0.567	91.80	0.000			
X_2_X_2_ (β_22_)	−0.249	17.76	0.003	0.247	13.66	0.004
X_3_X_3_ (β_33_)	−0.0003	20.30	0.002			
R^2^	0.948			0.704		
Adjusted R^2^	0.908			0.623		
Lack of fit		1.37	0.480		0.51	0.802
AAD (%)	2.760			5.581		

^a^ X_1_, X_2_, and X_3_ represent the NaCl concentration (% *w*/*v*), liquid-to-solid ratio (mL/g), and stirring time (min), respectively. ^b^ *p*-value more than 0.05 is not significantly different at a 5% level.

**Table 4:** A comparison between the experimental and predicted values for the response variables at optimal conditions of extraction yield and PV of silkworm pupae oil.

Response Variables	Experimental Value	Predicted Value	*p*-Value ^a^
Extraction yield (wt%)	3.32 ± 0.03	3.38	0.154
PV (mM)	1.55 ± 0.02	1.54	0.147

^a^ *p*-value more than 0.05 is not significantly different at a 5% level (one-sample *t*-test).

**Table 5:** The fatty acid composition, content of minor compounds, and DPPH free radical scavenging activity of silkworm pupae oil extracted by aqueous saline methods.

Criteria	Content
Fatty acid composition (% Total fatty acids)	
Saturated fatty acids	33.10 ± 0.05
Caprylic acid (C8:0)	0.12 ± 0.01
Lauric acid (C12:0)	0.05 ± 0.00
Myristic acid (C14:0)	0.16 ± 0.00
Palmitic acid (C16:0)	26.0 ± 0.05
Stearic acid (C18:0)	6.78 ± 0.01
Monounsaturated fatty acids (MUFA)	37.82 ± 0.03
Palmitoleic acid (C16:1)	0.98 ± 0.00
Oleic acid (C18:1)	36.84 ± 0.03
Polyunsaturated fatty acids (PUFA)	29.09 ± 0.07
Linoleic acid (18:2, n-6)	4.25 ± 0.01
Linolenic acid (18:3, n-3)	24.85 ± 0.06
Minor compounds	
Cholesterol (mg/100 g)	108.66 ± 0.09
β-Carotene (μg/100 g)	784.89 ± 12.17
α-Tocopherol (μg/100 g)	9434.39 ± 367.95
DPPH free radical scavenging activity (%)	57.59 ± 0.93

## Data Availability

The datasets generated for this study are available on request to the corresponding author.

## Supplementary Materials

The following are available online at https://www.mdpi.com/article/10.3390/foods11030291/s1, Equation (S1), Table S1. Peroxide values (PV) of silkworm pupae oil in mM and meq/kg oil.
