# Supplementary material for: Application of Aqueous Saline Process to Extract Silkworm Pupae Oil (Bombyx mori): Process Optimization and Composition Analysis
Source: Foods. 2022 Jan 21;11(3):291. doi: 10.3390/foods11030291 (PMC8834069; doi:10.3390/foods11030291)
Supplement: Supplementary file 1 [file foods-11-00291-s001.zip › foods-1529432-supplementary.pdf]

foods MDPI

Supplementary Material

Application of Aqueous Saline Process to Extract Silkworm Pupae Oil (*Bombyx mori*): Process Optimization and Composition Analysis

Janjira Tangsanthatkun [1,2], Methavee Peanparkdee [1,2], Wattinee Katekhong [1,2], Thepkunya Harnsilawat [2,3], Chin Ping Tan [4] and Utai Klinkesorn [1,2,*]

1 Department of Food Science and Technology, Faculty of Agro-Industry, Kasetsart University, 50 Ngam Wong Wan Road, Chatuchak, Bangkok 10900, Thailand; janjira_2u@hotmail.com (J.T.); methavee.pe@ku.ac.th (M.P.); fagiwnk@ku.ac.th (W.K.)
2 Research Unit on Innovative Technologies for Production and Delivery of Functional Biomolecules, Kasetsart University Research and Development Institute (KURDI), 50 Ngam Wong Wan Road, Chatuchak, Bangkok 10900, Thailand; thepkunya.h@ku.ac.th
3 Department of Product Development, Faculty of Agro-Industry, Kasetsart University, 50 Ngam Wong Wan Road, Chatuchak, Bangkok 10900, Thailand; thepkunya.h@ku.ac.th
4 Department of Food Technology, Faculty of Food Science and Technology, Universiti Putra Malaysia, 43400 Serdang, Selangor, Malaysia; tancp@upm.edu.my
* Correspondence: utai.k@ku.th

Equation S1.

$$Recovery\ oil\ (\%) = (Extracted\ oil\ weight / Initial\ oil\ content\ in\ sample) \times 100 \quad (1)$$

where initial oil content in sample is 6.07 g/100 g silkworm pupae for the current work.

Table S1. Peroxide values (PV) of silkworm pupae oil in mM and meq/kg oil.

Data sources	Peroxide value	
	mM	meq/kg oil
Standard value[a]‡	6.69[c]	15.00
Experiment value[b]	1.55	3.47

[a]Standard value according to Codex Alimentarius [1]; [b]Obtained from aqueous saline extraction of silkworm oil under optimal conditions; [c]Conversion of peroxide value in mM to meq/kg [2] using the following equation:

$$PV_1\ (meq/kg) = PV_2 (mM) \times (2/density)$$

where PV_1 and PV_2 are peroxide value expressed in meq/kg and mM, respectively. The correction factor of 2 (valence for O_2) is used for conversion of PV in meq to mmol. Density of aqueous saline silkworm oil of 0.8925 is used in the current work.

References

1. Alimentarius, C. Codex Standard for edible fats and oils not covered by individual standards. Codex Stan 1981, 19, 1-6.
2. International Fragrance Association. IFRA Analytical Method: Determination of the peroxide value. Available online: https://ifrafragrance.org/docs/default-source/guidelines/20190910-revised-ifra-analytical-method-on-peroxide-value.pdf?sfvrsn=c4a931e2_0 (accessed on 5 October 2021).
